# Deviations in continuously monitored electrodermal activity before severe clinical complications: a clinical prospective observational explorative cohort study

**DOI:** 10.1007/s10877-023-01030-4

**Published:** 2023-05-17

**Authors:** Andreas Ohrt Johansen, Jesper Mølgaard, Søren Straarup Rasmussen, Ying Gu, Katja Kjær Grønbæk, Helge B. D. Sørensen, Eske Kvanner Aasvang, Christian Sylvest Meyhoff

**Affiliations:** 1 Department of Anaesthesia and Intensive Care, Copenhagen University Hospital-Bispebjerg and Frederiksberg, Copenhagen, Denmark; 2 Department of Anaesthesiology, Centre for Cancer and Organ Dysfunction, Copenhagen University Hospital-Rigshospitalet, Copenhagen, Denmark; 3 Department of Health Technology, Technical University of Denmark, Kgs. Lyngby, Denmark; 4 Department of Clinical Medicine, University of Copenhagen, Copenhagen, Denmark

**Keywords:** Electrodermal activity, Galvanic skin response, Continuous monitoring, Wireless electronic devices, Vital signs, Physiological abnormalities

## Abstract

Monitoring of high-risk patients in hospital wards is crucial in identifying and preventing clinical deterioration. Sympathetic nervous system activity measured continuously and non-invasively by Electrodermal activity (EDA) may relate to complications, but the clinical use remains untested. The aim of this study was to explore associations between deviations of EDA and subsequent serious adverse events (SAE). Patients admitted to general wards after major abdominal cancer surgery or with acute exacerbation of chronic obstructive pulmonary disease were continuously EDA-monitored for up to 5 days. We used time-perspectives consisting of 1, 3, 6, and 12 h of data prior to first SAE or from start of monitoring. We constructed 648 different EDA-derived features to assess EDA. The primary outcome was any SAE and secondary outcomes were respiratory, infectious, and cardiovascular SAEs. Associations were evaluated using logistic regressions with adjustment for relevant confounders. We included 714 patients and found a total of 192 statistically significant associations between EDA-derived features and clinical outcomes. 79% of these associations were EDA-derived features of absolute and relative increases in EDA and 14% were EDA-derived features with normalized EDA above a threshold. The highest F1-scores for primary outcome with the four time-perspectives were 20.7–32.8%, with precision ranging 34.9–38.6%, recall 14.7–29.4%, and specificity 83.1–91.4%. We identified statistically significant associations between specific deviations of EDA and subsequent SAE, and patterns of EDA may be developed to be considered indicators of upcoming clinical deterioration in high-risk patients.

**Supplementary Information:**

The online version contains supplementary material available at 10.1007/s10877-023-01030-4.

## Introduction

Monitoring of patients’ vital parameters is important in early identification and prevention of clinical deterioration. Electrodermal activity (EDA) measurements are a potential supplement to vital signs measurements because it reflects sympathetic nervous activity [1]. With modern technology, EDA is a non-invasive measurement of electrical conductance on the surface of the skin with an easy-to-attach wristband [2]. It can be characterized to consist of two types of activity: a slowly varying baseline signal and a rapidly changing phasic activity, with the latter considered to be an index of sympathetic activity [3]. The magnitude of deviations in continuously measured EDA is, however, not well described, nor is their associations to subsequent patient complications.

Multiple studies have found that delayed identification of clinical deterioration is associated with avoidable intensive care unit admissions, serious adverse events (SAE), and in-hospital mortality [4–6]. Most frequent post-operative complications are pulmonary and circulatory diseases and for cardiovascular complications specifically it is estimated that 10 million patients experience myocardial injury after non-cardiac surgery each year [7, 8]. Chronic obstructive pulmonary disease is the third leading cause of death [9] and acute exacerbation of chronic pulmonary obstructive disease (AECOPD) patients have a mortality rate of 4–11% during hospital stay, and 21–43% during the first year [10, 11]. EDA measures the electrical conductance of the skin which is increased by perspiration. The sympathetic nervous system innervates sudomotor activity of the eccrine sweat glands [12] and thus EDA can be used as an indirect expression of sympathetic nervous system activity. EDA may identify underlying health conditions and alert for deterioration in the same way other vital parameters are used. This could particularly benefit patients because EDA is easy to measure continuously in real-time.

As there are no well-established thresholds for normal EDA, we aimed to define and explore a large variety of associations between EDA-derived features and subsequent SAEs. We conducted a hypothesis-generating study to determine which deviations of EDA that had the strongest association with subsequent SAE.

## Materials and methods

### Study setting

This project was a part of the WARD project (Wireless Assessment of Respiratory and circulatory Distress), which aims to develop artificial intelligence algorithms to detect patient deterioration in real-time vital sign monitoring. Data for this prospective observational cohort study was collected at Bispebjerg Hospital and Rigshospitalet, Copenhagen, Denmark between February 2018 and August 2020 in two study populations: WARD-COPD (H-18026653, NCT03660501) and WARD-Surgery (H-17033535, NCT03491137). The patient population consisted of patients admitted with acute exacerbation of chronic obstructive pulmonary disease (AECOPD) and surgery patients. The surgical patient population was divided into four groups with different surgical procedures: colorectal cancer surgery, surgery involving the oesophagus and ventricle, surgery involving the pancreas (including the Whipple-procedure), and other surgical procedures. Inclusion criteria were: Adult patients (≥ 18 years of age) hospitalized with suspected acute exacerbation of COPD or patients (≥ 60 years of age) admitted for major abdominal cancer surgery (e.g., colonic resections, gastrectomy, pancreatic resections) with estimated duration of surgery ≥ 2 h and at least two expected overnight stays. Exclusion criteria were: Patient expected not to cooperate with study procedures, allergy to plaster or silicone, impaired cognitive function (assessed by a Mini Mental State Examination score < 24), any treatment limitation (such as a ‘do-not-resuscitate’ order), a pacemaker or implantable cardioverter defibrillator device, and for AECOPD patients: expected admittance < 24 h.

### Data sources

EDA was monitored using Empatica E4 wristband (Empatica Inc., Boston, USA) with a sampling frequency of four EDA measurements per second. Patients were monitored continuously until discharge or for a maximum of five days. Study personnel made daily visits to verify correct placement of equipment, change batteries, and ensure compliance. The EDA recordings were not visible for patients, clinical, or research personnel during the study period. Patients were also monitored continuously on the following parameters with the following devices: Heart rate was measured continuously using Isansys Life Touch patch placed on the chest (Isansys Lifecare, Oxfordshire, United Kingdom). Non-invasive arterial blood pressure was measured every 30 min from 07:00 to 22:00, and every 60 min from 22:00 to 7:00, using BlueBP (Meditech, Budapest, Hungary). This data was also blinded to clinicians.

### Exposure variables

We defined and explored a wide variety of EDA-derived features as exposure variables. The amplitude of the phasic activity was the primary diagnostic feature of EDA [13], and thus the EDA amplitude was chosen as primary exposure variable (Table 1). We focused on the EDA data measured within 1, 3, 6, and 12 h prior to SAE with the primary focus on 6 h prior to complication. For patients without SAEs, we extracted the first 12 h of available EDA monitoring data and used 1, 3, 6, and all 12 h of the data depending on the time-perspective. The EDA amplitude was measured in micro siemens (μS) and we predefined 648 different patterns of EDA-derived features to assess EDA, in eight different categories, for the purpose of isolating the rapidly changing phasic activity component of EDA. The first category was through statistical values (e.g., average EDA). The second category was to focus on the cumulated number of EDA measurements with EDA above a certain threshold. The third category was to focus on deviations with EDA above a certain threshold through the duration of a certain time, for at least 90% of the time. The fourth category was to normalize the EDA axis to values between 0 and 1 and focus on the cumulated number of measurements of EDA with EDA above certain thresholds. The fifth category was to normalize the EDA axis to values between 0 and 1 and focus on deviations with EDA above a certain threshold through the duration of a certain time, for at least 90% of the time. To prevent artefact EDA measurements of large amplitude from affecting the normalized EDA data, we set an EDA ceiling for each dataset with the value just below the top 1% of EDA measurements and changed the top 1% to this ceiling value. The sixth category was to focus on deviations of an absolute increase in EDA within a timeframe. The seventh category was to focus on deviations of a relative increase in EDA (for EDA values above 1) within a timeframe. The eighth category was to perform a Fast Fourier Transformation (FFT) and compare the spectral analysis graphs. The frequencies 0.045–0.25 Hz are the most sensitive to central sympathetic control at rest [14, 15] and therefore were the frequencies we primarily wanted to analyse for predictors of serious adverse complications. The spectral analyses were normalized. The Nyquist Theorem states that an EDA sampling frequency of 4 measurements per second can produce a spectral analysis of up to 2 Hz maximum resolution. The EDA data was split into one-hour segments and analysed individually. We used the cumulated amplitude of multiple one-hour segment analyses of the frequency bands.

### Secondary exposure variables

Deviations of heart rate and blood pressure were secondary exposure variables because these are usual vital sign indicators of circulatory imbalance. These were both using 12 h of data (prior to SAE or after monitoring start). Tachycardia deviations were defined as a heart rate > 111 bpm for ≥ 60 consecutive minutes and a heart rate > 130 bpm for ≥ 30 consecutive minutes [16]. Hypotension deviations were defined as systolic blood pressure ≤ 90 mmHg for two consecutive measurements and a systolic blood pressure ≤ 70 mmHg for one consecutive measurement.

### Outcomes

The primary outcome in this study was any SAE, according to the International Council for Harmonisation of Technical Requirements for Pharmaceuticals for Human Use – Good Clinical Practice (ICH-GCP), occurring within the time of continuous monitoring. The three secondary outcomes were SAEs of infectious, respiratory or cardiovascular aetiology. Serious adverse infectious events were defined as one of the following events: Urinary tract infection, sepsis, septic shock, surgical site infection, or pneumonia evaluated to be a SAE according to ICH-GCP. Serious adverse respiratory events were defined as one of the following events: Respiratory failure (defined as requirement for non-invasive or mechanical ventilation or high-flow oxygen therapy), pneumonia, atelectasis, pneumothorax, or pleural effusion evaluated to be a serious adverse event according to ICH-GCP. Serious adverse cardiovascular event were defined as one of the following events, if they were categorized as SAE according to ICH-GCP: Pulmonary embolism, new onset heart failure, non-fatal cardiac arrest, troponin elevation of ischemic aetiology, myocardial infarction, new onset atrial fibrillation or flutter, other supraventricular tachyarrhythmias, ventricular tachycardia, new onset second degree atrio-ventricular block, or new onset third degree atrio-ventricular block, all within the time of continuous monitoring. If patients had more than one SAE, only the first was used in the data-analysis.

### Adjustment variables

The adjustment variables were patient group (AECOPD, type of surgery (colorectal, oesophagus/ventricle, pancreas, and other), age (above/below 70 years) and Charlson Comorbidity Index (CCI).

### Data processing

**Fig. 1:**
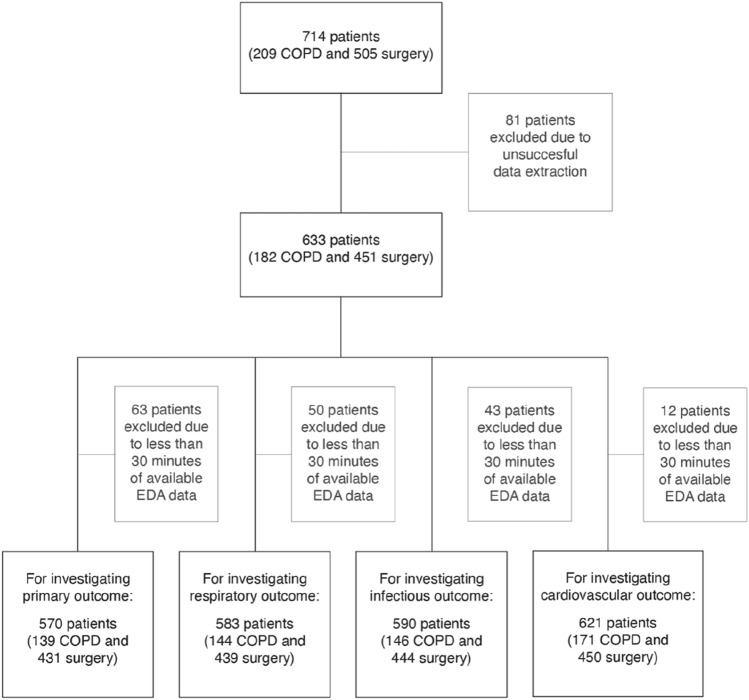
Patient flowchart

We calculated two variables to identify the amount of missing data in the EDA data of each patient: the accumulated gaps of missing data in the monitoring (e.g., if the battery runs out) and the lack of monitoring time if an SAE occurs before we had the full 12 h of monitoring prior to SAE. Dividing the expected time of monitoring (1, 3, 6, or 12 h) with the available monitoring time we calculated a scaling factor, which was used to normalize the data. Patients with less than 30 min of total monitoring were excluded (Fig. 1). In the processed electrodermal data, we detected the primary exposure variables of EDA (consisting of statistical values, patterns of changes in EDA, and spectral analyses) and secondary exposure variables of tachycardia and hypotension. Multiple detections of the same deviation did not overlap on a timeline of a patients EDA data (e.g., the algorithm did not detect multiple instances of a deviation of EDA ≥ 10 μS for 2 min in 3 min of EDA data above 10 μS).

### Statistical analyses

Logistic regression analyses were performed to calculate the odds ratio (OR) of the associations between the independent variables (statistical values, patterns of changes in EDA, and amplitude of frequency bands) and whether the patient experienced a serious adverse event or not (dependent binary variable), adjusted for the three adjustment variables. We split the data into a training set and a test set in order to optimize clinical relevancy and calculate precision, recall/sensitivity, specificity, and F1-score. The split was stratified according to the three adjustment variables. Using the training set a number of associations were identified. The associations with P-values < 0.05 were considered statistically significant and were evaluated on the testing set. Furthermore, only the associations with the 95% confidence interval of the OR being above 1.00 were evaluated on the testing set as to ensure identification of only EDA-derived features that increase the risk of subsequent SAE. The data was split into a training set and testing with 25 and 75% of the data, respectively. Statistical analyses were performed in Python (version 3.8, Python Software Foundation, Delaware, USA). To further evaluate the validity of the results, a comparison to a random classifier was conducted. A random classifier was created for each of the four outcomes. The classifier randomly guessed SAE or not SAE with a predetermined chance and for a number of times matching the number of patients in the test sets. The chance to guess SAE was based on the prevalence of SAE in the test sets for each outcome. For each classifier the calculations were repeated 200 times and the average values including 95% confidence intervals and F1-score comparisons were calculated.

The EDA-derived features with the strongest associations to the outcomes, measured as highest F1-score, were also compared to the best associations between the secondary exposure variables (tachycardia and hypotension) and study outcomes.

## Results

Five-hundred-seventy patients were included in the primary analysis (Fig. 1), of which the mean age was 71, 59% were men, 93% had a CCI of 3 and above, and a total of 156 patients (27%) had an SAE occurring within the time of monitoring (Table 2). The total monitoring time was 6214 h and EDA data was available for 99.34% of the time. The analysis of respiratory, infectious, and cardiovascular outcomes respectively had 583, 590, and 621 patients (Fig. 1). For the secondary outcomes, 77 (13%), 38 (6.4%), and 71 (11%) had a respiratory, infectious, and cardiovascular outcome within the time of monitoring, respectively. A total of 648 patterns of EDA-derived features were defined in the eight categories (Table 1). The analysis of time-perspectives (1, 3, 6, and 12 h of EDA data prior to SAE or since start of monitoring) and four outcomes, resulted in 10,368 potential associations between EDA and subsequent SAE.

We found a total of 192 statistically significant associations in the 10,368 potential associations, with the associations to the primary outcome having a maximal F1-score of 32.8%. Forty-seven (22%) of all the associations were to the primary outcome, 43 (21%) with respiratory outcome, 64 (31%) with infectious outcome, and 38 (18%) with cardiovascular outcome. The majority of the significant associations were in one of two similar categories of EDA-derived features. The category of relative EDA increases within a timeframe held 86 (45%) associations and the category of absolute EDA increases within a timeframe held 66 (34%) associations. EDA-derived features of EDA values above a threshold for a duration of certain time held 13 (6.8%) associations and EDA-derived features of normalized EDA values above a threshold for a duration of certain time held 26 (14%) associations.

The secondary analyses of tachycardia produced two statistically significant associations with subsequent cardiovascular outcome: a heart rate above 130 for 30 min was associated with cardiovascular outcome within 6 h and a heart rate above 111 for 60 min was associated with cardiovascular outcome within 12 h. The secondary analyses of hypotension produced no statistically significant associations with any of the outcomes.

The distribution of all statistically significant associations between EDA-derived features and the four outcomes was presented in Table 3. For each outcome and time-perspective, we have presented the associations with the highest F1-scores (Table 4). The secondary analyses were presented (Table 5). All patients were monitored in hospital wards and did not receive vasopressors or anticholinergic medications.

The random classifier had F1-scores with 95% confidence intervals for primary outcome 25.32% (24.78–25.86%), for respiratory outcome 13.84% (13.24–14.43%), for infectious outcome 6.03% (5.38–6.68%), and for cardiovascular outcome 11.53% (10.98–12.09%). These were compared to the performance of the EDA-derived features and presented in Supplementary Appendix Table 1.

## Discussion

In this large hypothesis-generating observational study, we detected several different EDA-derived features and found statistically significant associations between EDA-derived features and subsequent severe clinical complications. The most dominant exposure variables were EDA-derived features of relative and absolute increases within a timeframe, as they accounted for 79% of all statistically significant associations with the outcomes, indicating a potentially optimal method of measuring phasic EDA without the tonic baseline EDA. The two categories of both EDA and normalized EDA above a threshold for a duration of certain accounted for 20% of all significant associations. These two categories were methodically similar as they quantified EDA-derived features as plateaus or spikes with EDA values above absolute thresholds. This demonstrated that elevated EDA values can be associated with subsequent clinical deterioration. The spectral analyses did not produce any significant associations and was not considered an optimal way of assessing EDA.

Across all four time-perspectives, the highest F1-scores were found in association to the primary outcome and ranging 20.7–32.8%, with precision ranging 34.9–38.6%, recall ranging 14.7–29.4%, and specificity ranging 83.1–91.4%. For the all outcomes with the time-perspectives of 1, 3, and 6 h the EDA-derived features of absolute and relative increases in EDA had the highest F1-scores. This indicates EDA-derived features of relative and absolute increases within a timeframe as an optimal approach when monitoring for 1–6 h. With the time-perspective of 12 h the EDA-derived features of normalized EDA above a threshold with the highest F1-scores for primary, infectious, and cardiovascular outcomes. All 26 statistically significant associations of normalized EDA above a threshold were with a 12-h time-perspective. This indicates a potential relevancy of individually calibrated EDA thresholds when monitoring for 12 h. The EDA-derived features of EDA values above absolute thresholds held only 13 statistically significant associations. The use of absolute values of EDA was affected by an impediment to error in measurement due to its dependence on factors such as ambient environment and electrode size [17]. We were not controlling for how tight the device was worn.

The performance of secondary exposure of tachycardia was within the range of the performance of the EDA-derived features, but not surpassing it. The tachycardia was only significantly associated with cardiovascular outcomes, with F1-scores ranging 5.1–5.9%, precision 15.4–33.3%, recall 3.1–3.2%, and specificity 97.3–99.0%. For the same outcome the EDA-derived features had F1-scores ranging 3.4–6.8%, precision 14.3–25.0%, recall 1.8–4.4%, and specificity 97.2–99.3%. Tachycardia is well-established as being associated with an increased risk of SAE and mortality in post-operative patients [18, 19] and this argued for the relevancy of EDA-derived features as a utility in patient monitoring. The secondary outcome of hypotension was not statistically significantly associated any outcomes.

In this study we investigated a large number of EDA-derived features in eight different categories. A study from University of Chicago that included 269,999 medical and surgical admissions in five hospitals in Illinois for identifying critical illness in patients using vital sign trends [20]. The exposure variables were six vital signs assessed by seven trajectories: current value, minimum, maximum, mean, standard deviation, delta, slope, and smoothing. The primary outcome was a composite outcome of cardiac arrest, intensive care unit transfer, and death. They concluded a significantly increased accuracy in detecting clinical deterioration when using these different trajectories compared to only using current values. This argues for our methodology of using multiple categories of EDA-derived features. Another study sought to investigate whether temperature measured at the wrist with an Empatica E4 could accurately identify patients with infections [21]. They used 88 recording sessions of patients retrospectively classified in three groups as either having no infection (60 datasets) or having an infection with either less or more than 24 h of antibiotics treatment (5 and 23 datasets, respectively). Patients with infections had a significantly higher mean EDA value, which supports our findings of infectious outcome mostly having the highest precision values when using the EDA-derived features of EDA values above a threshold.

A study from the University of California investigated the utility of EDA as a pain assessment tool for post-operative pain in 25 adult patients [22]. They analysed EDA through pre-processing consisting of down-sampling, moving average smoothing, and normalization, then a Python library, with the name pyEDA [23], was used for feature extraction. They found the most important features of EDA in pain assessment to be the maximum value and root mean square of the EDA signal. This is a different approach than what we have demonstrated in this paper, in identifying associations between EDA-derived features and an outcome, and it demonstrates that EDA is an area still in a state of experimentation with no well-established definition of what range of EDA is considered pathological or otherwise abnormal.

We used wireless Empatica E4 wristbands in our study to monitor EDA, and a study found that the Empatica E4 produced unreliable EDA data when this device was compared to the MindWare device [24]. The MindWare device measures EDA with a sampling rate of 500 Hz using two wired electrodes placed on the palmar manus, with a high density of eccrine glands [25]. It is important to find a balance between how much accuracy is necessary to reliably detect clinical outcomes and how much practicality (e.g., battery life) and ease-of-use is necessary for the technology to be applicable for patient monitoring. Our results of the utility of EDA are found using the Empatica E4, and in order to further verify the utility of EDA in patient monitoring, further studies are essential in determining the most accurate methods and optimal devices. A Norwegian study found both the number and the amplitude of very small increases in EDA (as small as 0.02 micro siemens) were positively correlated with blood pressure and epinephrine levels in the blood during laparoscopic cholecystectomy [26]. This is similar to our methodology of detecting the accumulated number of EDA-derived features and argues in favor of our main hypothesis.

The basis for this hypothesis-generating study is founded on plausible associations due to a well-documented scientific link from sympathetic nerve innervation of sweat glands to EDA being an index of skin surface perspiration. This study is a thorough investigation of EDA taking numerous exposure variables into account as well as adjustment variables. We have a large patient population of 714 patients, both medical and post-surgery patients, resulting in a broad generalizability to the high-risk patients in general hospital wards.

This study also comes with limitations. First, the main limitation is the issue of statistical multiplicity. We tested 648 different associations simultaneously and as a result had a substantial increased risk of type I errors. Different statistical methods exist to correct for type I errors when testing multiple hypotheses simultaneously, e.g., Bonferroni corrections. We decided a priori not to use corrections for statistical multiplicity, as we wanted to avoid type II errors closing off potentially constructive domains of research [27]. Our purpose was solely hypothesis generation, and not to draw firm clinical conclusions. Second, we included high-risk patients, which may have introduced confounders in the association between EDA-derived features and clinical complications. Patients were either AECOPD patients or post-operative after major surgery, and most had other comorbidities (only 7% of the patients in the primary outcome population had a CCI of 2 or less). To assess a clear signal in the association between EDA-derived features and subsequent clinical complication, we only analysed EDA data until the first SAE in each patient. Third, the EDA recording had periods of missing data. We compensated for datasets with less than 12 h of data by normalizing the length of the datasets to match the datasets with all 12 h of data. However, periods of missing data were not taken into consideration on a chronological timeline, as they were removed from the datasets. This could cause distortions in the timeline and it could affect the spectral band analyses, which could explain why there were no statistically significant associations between EDA spectral bands and subsequent SAE. An attempt was made to prevent this issue as we performed FFT on one-hour data segments. Our study aimed to identify the patterns of EDA-derived features with the highest precision and recall for subsequent serious complications and should therefore be interpreted with caution as explorative analyses. Their validity should be tested in future studies.

After additional validation comparison to a random classifier, the results argued for the optimal performance being in association to primary outcome and infectious outcome. It also argued in favor of the associations capturing a true signal.

EDA may provide additional important clinical information in combination with traditional vital sign monitoring such as heart rate and pulse oximetry. In the future, we suggest an EDA-algorithm that functions as an add-on to the existing patient monitoring system and improving the accuracy through working with multiple patterns of EDA-derived features, continuously calculating an at-risk score and relaying an alarm to the medical personnel when this score is above a certain threshold for risk of severe clinical complications. Further studies are needed to investigate how EDA performs when used in combination with other vital parameters and whether the addition of EDA monitoring can improve performance in detection of clinical deterioration.

## Conclusion

We identified statistically significant associations between EDA-derived features and subsequent SAE with all four outcomes and all four time-perspectives. The EDA-derived features of absolute and relative increases constituted 79% of all statistically significant associations and had the highest F1-scores across all four outcomes and the time-perspectives of 1, 3, and 6 h, and as a result can be indicators of subsequent clinical deterioration in high-risk patients. Using a 12-h time-perspective we also identified the EDA-derived features of normalized EDA above a threshold as statistically significant and having the highest F1-scores in association with primary outcome, infectious outcome, and cardiovascular outcome. This indicates a potential relevancy of individually calibrated EDA thresholds when monitoring for 12 h or more. A comparison to a random classifier suggested optimal performance of EDA-derived features in association to primary outcome and infectious outcome.

### Electronic supplementary material

Below is the link to the electronic supplementary material.

Supplementary file1 (DOCX 20 kb)

## Appendix

See Fig. 1 and Tables 1, 2, 3, 4 and 5.

**Table 1:** A table of the eight groups of EDA-derived features for use as exposure variables

**(1) Statistical values (4 variables)** Average EDA, 25th percentile, 50th percentile/the median, and the 75th percentile
**(2) Cumulated time of EDA above a certain threshold (15 variables):**
EDA ≥ 2 μS, 4 μS, 6 μS, 8 μS, 10 μS, 15 μS, 20 μS, 30 μS, 40 μS, 50 μS, 60 μS, 80 μS, 100 μS, 150 μS, and 200 μS
**(3) EDA above a certain threshold for the duration of a certain time (150 variables)**
EDA ≥ 2 μS, 4 μS, 6 μS, 8 μS, 10 μS, 15 μS, 20 μS, 30 μS, 40 μS, 50 μS, 60 μS, 80 μS, 100 μS, 150 μS, and 200 μS. For the duration ≥ 1 s, 5 s, 10 s, 20 s, 30 s, 1 min, 2 min, 3 min, 5 min, and 10 min
**(4) Cumulated time of normalized EDA above a certain threshold (10 variables):**
EDA ≥ 0.1, 0.2, 0.3, 0.4, 0.5, 0.6, 0.7, 0.8, 0.9, and 0.95
**(5) Normalized EDA above a certain threshold for the duration of a certain time (100 variables)**
EDA ≥ 0.1, 0.2, 0.3, 0.4, 0.5, 0.6, 0.7, 0.8, 0.9, and 0.95. For the duration ≥ 1 s, 5 s, 10 s, 20 s, 30 s, 1 min, 2 min, 3 min, 5 min, and 10 min **(6) An increase in EDA by an absolute value within a timeframe (150 variables** EDA increases by 1 μS, 2 μS, 3 μS, 4 μS, 5 μS, 6 μS, 8 μS, 10 μS, 15 μS, 20 μS, 30 μS, 40 μS, 50 μS, 75 μS, and 100 μS. Within the timeframe of 1 s, 5 s, 10 s, 20 s, 30 s, 1 min, 2 min, 3 min, 5 min, and 10 min
**(7) An increase in EDA by a relative value within a timeframe (150 variables)**
EDA (for EDA values above 1) increases in value of 50%, 100%, 200%, 300%, 400%, 500%, 600%, 800% 1000%, 2000%, 3000%, 4000%, 5000%, 7500%, and 10,000%. Within the timeframe of 1 s, 5 s, 10 s, 20 s, 30 s, 1 min, 2 min, 3 min, 5 min, and 10 min
**(8) Fourier transformation spectral analysis (69 variables)**
The amplitude of 9 frequency bands with a width of 0.205 Hz beginning from 0.045 Hz to 1.830 Hz. (0.045 Hz–0.250 Hz, 0.250 Hz–0.455 Hz, etc.) and the amplitude of 60 frequency bands with a width of 0.005 Hz beginning from 0.005 Hz to 0.305 Hz. (0.005 Hz–0.010 Hz, 0.010 Hz–0.015 Hz, etc.)

**Table 2:** Baseline characteristics of the primary outcome population

	AECOPD patients (N = 139)	Surgery patients (N = 431)	Total (N = 570)
Male sex, n (%)	64 (46.0%)	272 (63.1%)	336 (58.9%)
Age (years), mean (SD)	73 (± 11)	71 (± 6.2)	71 (± 7.7)
Body mass index (kg/m2), mean (SD)*	25 (± 6.1)	26 (± 4.4)	26 (± 4.8)
Medical history as Charlson Comorbidity Index, n (%)			
0–2	13 (9.4%)	27 (6.3%)	40 (7.0%)
3–5	81 (58.3%)	268 (62.2%)	349 (61.2%)
6–8	40 (28.8%)	111 (25.8%)	151 (26.5%)
9–11	5 (3.6%)	23 (5.3%)	28 (4.9%)
12–15	0 (0%)	2 (0.5%)	2 (0.4%)
Systolic BP (mmHg), mean (SD)*	140 (± 23)	140 (± 19)	140 (± 20)
Diastolic BP (mmHg), mean (SD)*	72 (± 13)	76 (± 11)	75 (± 12)
Peripheral oxygen saturation (%), mean (SD)*	94 (± 2.6)	98 (± 1.8)	97 (± 2.6)
SAE within time of monitoring, n (%)	41 (29.5%)	115 (26.7%)	156 (27.4%)
Hours of EDA data,	12.0 [0.504, 12.0]	12.0 [0.624, 12.0]	12.0 [0.504, 12.0]
Median [Min, Max]			
< 3 h of data, n (%)	21 (15.1%)	12 (2.8%)	33 (5.8%)
< 1 h of data, n (%)	11 (7.9%)	5 (1.2%)	16 (2.8%)
Reason for admission, n (%)			
AECOPD	139 (100%)	0 (0%)	139 (24.4%)
Colorectal surgery	0 (0%)	145 (33.6%)	145 (25.4%)
Esophagus/Ventricle surgery	0 (0%)	114 (26.5%)	114 (20.0%)
Pancreatic surgery	0 (0%)	154 (35.7%)	154 (27.0%)
Other surgery	0 (0%)	18 (4.2%)	18 (3.2%)
American Society of Anaesthesiologists’ class (I/II/III/IV), n (%)		21 (4.9%) /	
		232 (53.8%) /	
		175 (40.6%) /	
		3 (0.7%)	
Global initiative for chronic obstructive lung disease score (GOLD 1/2/3/4), n (%)	15 (10.8%) /		
	36 (25.9%) /		
	56 (40.3%) /		
	0 (0%)		
Missing values	32 (23.0%)		
Smoking status as current smoker, n (%)	56 (40.3%)	57 (13.2%)	113 (19.8%)
Alcohol consumption above recommended, n (%)	24 (17.3%)	84 (19.5%)	108 (18.9%)

*Less than 2.5% missing data

**Table 3:** Distribution of statistically significant associations in the training data

Time perspective (hours)	(1) Statistical values	(2) Cumulated time of EDA above a certain threshold	(3) EDA above a certain threshold for the duration of a certain time	(4) Cumulated time of normalized EDA above a certain threshold	(5) Normalized EDA above a certain threshold for the duration of a certain time	(6) An increase in EDA by an absolute value within a timeframe	(7) An increase in EDA by a relative value within a timeframe	(8) Fourier transformation spectral analysis	Total n. (%)
1	0	0	0	0	0	3	7	0	10 (5.2%)
3	0	0	0	0	0	7	10	0	17 (8.9%)
6	0	0	0	0	0	2	2	0	4 (2.1%)
12	0	0	1	0	13	1	1	0	16 (8.3%)
1	0	0	0	0	0	5	10	0	15 (7.8%)
3	0	0	0	0	0	8	10	0	18 (9.4%)
6	0	0	1	0	0	3	3	0	7 (3.6%)
12	0	0	0	0	1	2	0	0	3 (1.6%)
1	0	0	0	0	0	3	6	0	9 (4.7%)
3	0	0	1	0	0	4	15	0	20 (10.4%)
6	0	0	2	0	0	5	7	0	14 (7.3%)
12	0	0	0	0	9	3	9	0	21 (10.9%)
1	1	0	1	0	0	8	0	0	10 (5.2%)
3	0	0	1	0	0	8	0	0	9 (4.7%)
6	0	0	1	0	0	1	5	0	7 (3.6%)
12	0	0	5	0	3	3	1	0	12 (6.3%)
Total n. (%)	1 (0.5%)	0 (0.0%)	13 (6.8%)	0 (0.0%)	26 (13.5%)	66 (34.4%)	86 (44.8%)	0 (0.0%)	192 (100.0%)

Primary outcome of any SAE includes the following complications when classified as SAE: Mortality, admission to intensive care unit, delirium, syncope, stroke, transient ischemic attack, and any of the infectious, respiratory and cardiovascular outcomes

Infectious outcomes: Any infectious serious adverse event (one of the following: Urinary tract infection, sepsis, septic shock, surgical site infection, or pneumonia)

Respiratory outcomes: Any respiratory serious adverse event (one of the following: Respiratory failure, pneumonia, atelectasis, pneumothorax, or pleural effusion)

Cardiovascular outcomes: Any cardiovascular serious adverse event (one of the following: Pulmonary embolism, new onset heart failure, non-fatal cardiac arrest, troponin elevation of ischemic aetiology, myocardial infarction, new onset atrial fibrillation or flutter, other supraventricular tachyarrhythmias, ventricular tachycardia, new onset second degree atrio-ventricular block, or new onset third degree atrio-ventricular block)

**Table 4:** The EDA-derived features with the highest F1-scores evaluated on the test data

Outcome	Time perspective	Description	OR*	P-value*	Precision (%)	Recall (%)	Specificity (%)	F1-score (%)
Primary outcome	1	Increase by 800% within 5 min	11.86 (1.23–114.55)	0.033	38.46	22.94	87.46	28.74
	3	Increase by 50% within 10 min	1.08 (1.002–1.17)	0.045	37.21	29.36	83.07	32.82
	6	Increase by 100% within 30 s	1.03 (1.005–1.06)	0.022	34.88	14.71	91.41	20.69
	12	Normalized EDA above 0.95 for 30 s	1.02 (1.003–1.04)	0.022	38.60	19.47	88.89	25.88
Respiratory outcome	1	Increase by 200% within 3 min	4.05 (1.94–8.46)	0.0002	20.00	9.68	93.62	13.04
	3	Increase by 100% within 5 min	1.1 (1.01–1.19)	0.025	41.67	9.09	98.17	14.93
	6	Increase by 100% within 5 min	1.06 (1.01–1.12)	0.012	40.00	3.18	99.20	5.88
	12	Increase by 15 µS within 2 min	1.21 (1.01–1.44)	0.035	23.08	4.76	97.33	7.90
Infectious outcome	1	Increase by 100% within 5 min	1.47 (1.06–2.04)	0.022	50.00	6.25	99.51	11.11
	3	Increase by 500% within 10 min	2.17 (1.23–3.84)	0.007	13.33	6.67	96.85	8.89
	6	Increase by 200% within 10 min	1.31 (1.08–1.6)	0.007	33.33	8.57	98.53	13.64
	12	Normalized EDA above 0.8 for 30 s	1.01 (1.001–1.01)	0.011	60.00	11.11	99.52	18.75
Cardio-vascular outcome	1	Increase by 3 µS within 5 s	1.08 (1.01–1.15)	0.022	25.00	1.82	99.27	3.39
	3	Increase by 15 µS within 5 min	1.52 (1.11–2.08)	0.009	20.00	3.51	98.04	5.97
	6	Increase by 4000% within 5 min	13.99 (1.25–156.64)	0.032	16.67	1.89	98.79	3.39
	12	Normalized EDA above 0.4 for 3 min	1.01 (1.001–1.01)	0.027	14.29	4.44	97.15	6.78

*Values from the training data sets

**Table 5:** The secondary exposure variables of heart rate and blood pressure

Outcome	Time perspective	Exposure variable	OR*	P-value*	Precision	Recall	Specificity	F1-score
Cardio-vascular	6	Heart rate above 130 for 30 min	1.93 (1.13—3.28)	0.0155	33.3%	3.2%	99.0%	5.9%
	12	Heart rate above 111 for 60 min	1.31 (1.05—1.63)	0.0162	15.4%	3.1%	97.3%	5.1%

*Values from the training data sets
