# Supplementary material for: Deviations in continuously monitored electrodermal activity before severe clinical complications: a clinical prospective observational explorative cohort study
Source: J Clin Monit Comput. 2023 May 17;37(6):1573–84. doi: 10.1007/s10877-023-01030-4 (PMC10651525; doi:10.1007/s10877-023-01030-4)
Supplement: Supplementary file 1 — Supplementary file1 (DOCX 20 kb) [file 10877_2023_1030_MOESM1_ESM.docx]

**Supplementary Appendix**

**Table 1: Comparison of the EDA-derived features with the highest F1-scores to random classifier**

| Outcome | Time Perspective | Description | Precision | Recall | Specificity | F1-score | ΔF1-score* |
| --- | --- | --- | --- | --- | --- | --- | --- |
| Primary outcome | 1 | Increase by 800% within 5 minutes | 38,46% | 22,94% | 87,46% | 28,74% | 3,41% (2,87% - 3,96%) |
|  | 3 | Increase by 50% within 10 minutes | 37,21% | 29,36% | 83,07% | 32,82% | 7,5% (6,96% - 8,04%) |
|  | 6 | Increase by 100% within 30 seconds | 34,88% | 14,71% | 91,41% | 20,69% | -4,63% (-5,17% - -4,09%) |
|  | 12 | Normalized EDA above 0.95 for 30 seconds | 38,60% | 19,47% | 88,89% | 25,88% | 0,56% (0,02% - 1,1%) |
|  | Random classifier | | 25,37% (24,85% - 25,89%) | 25,37% (24,76% - 25,98%) | 74,19% (73,85% - 74,53%) | 25,32% (24,78% - 25,86%) |  |
| Respiratory outcome | 1 | Increase by 200% within 3 minutes | 20,00% | 9,68% | 93,62% | 13,04% | -0,79% (-1,39% - -0,19%) |
|  | 3 | Increase by 100% within 5 minutes | 41,67% | 9,09% | 98,17% | 14,93% | 1,09% (0,49% - 1,69%) |
|  | 6 | Increase by 100% within 5 minutes | 40,00% | 3,18% | 99,20% | 5,88% | -7,95% (-8,55% - -7,36%) |
|  | 12 | Increase by 15 µS within 2 minutes | 23,08% | 4,76% | 97,33% | 7,90% | -5,94% (-6,54% - -5,34%) |
|  | Random classifier | | 13,96% (13,37% - 14,56%) | 13,82% (13,19% - 14,45%) | 87% (86,76% - 87,25%) | 13,84% (13,24% - 14,43%) |  |
| Infectious outcome | 1 | Increase by 100% within 5 minutes | 50,00% | 6,25% | 99,51% | 11,11% | 5,08% (4,43% - 5,73%) |
|  | 3 | Increase by 500% within 10 minutes | 13,33% | 6,67% | 96,85% | 8,89% | 2,86% (2,21% - 3,51%) |
|  | 6 | Increase by 200% within 10 minutes | 33,33% | 8,57% | 98,53% | 13,64% | 7,6% (6,95% - 8,26%) |
|  | 12 | Normalized EDA above 0.8 for 30 seconds | 60,00% | 11,11% | 99,52% | 18,75% | 12,72% (12,07% - 13,37%) |
|  | Random classifier | | 6,12% (5,46% - 6,78%) | 6,06% (5,39% - 6,72%) | 94% (93,84% - 94,16%) | 6,03% (5,38% - 6,68%) |  |
| Cardiovascular outcome | 1 | Increase by 3 µS within 5 seconds | 25,00% | 1,82% | 99,27% | 3,39% | -8,14% (-8,7% -  -7,59%) |
|  | 3 | Increase by 15 µS within 5 minutes | 20,00% | 3,51% | 98,04% | 5,97% | -5,56% (-6,12% - -5,01%) |
|  | 6 | Increase by 4000% within 5 minutes | 16,67% | 1,89% | 98,79% | 3,39% | -8,14% (-8,7% -  -7,59%) |
|  | 12 | Normalized EDA above 0.4 for 3 minutes | 14,29% | 4,44% | 97,15% | 6,78% | -4,75% (-5,31% - -4,2%) |
|  | Random classifier | | 11,47% (10,93% - 12,02%) | 11,7% (11,1% - 12,3%) | 87,93% (87,69% - 88,17%) | 11,53% (10,98% - 12,09%) |  |

* The difference in F1-score was calculated as the F1-score of the EDA-derived features subtracted the F1-score 95% confidence interval of the random classifier to the same outcome.
